# Experimental and simulation study on the delayed release of CO in the initial stage of the low-temperature oxidation of coal

**DOI:** 10.1038/s41598-022-11120-z

**Published:** 2022-10-17

**Authors:** Zongxiang Li, Song Wei, Cong Ding, Mingqian Zhang, Zhibin Yang, Wenqing Wang

**Affiliations:** 1 College of Safety Science & Engineering, Liaoning Technical University, Fuxin, 123000 Liaoning China; 2 Research Institute of Safety Science and Engineering, Liaoning Technical University, Fuxin, 123000 Liaoning China; 3 Key Laboratory of Mine Thermal Disaster and Prevention, Ministry of Education (Liaoning Engineering Technology University), Fuxin, 123000 Liaoning China

**Keywords:** Environmental sciences, Chemistry, Materials science

## Abstract

To investigate the delayed release characteristics of CO gas in the initial stage of the low-temperature oxidation of coal, closed oxygen consumption experiments were conducted on coal samples taken from the Hongqingliang coal mine, and the corresponding relationship between the CO concentration and time in the initial stage of the experimental reaction was analyzed. A physical adsorption model of the macromolecules in coal for O_2_ and CO was established, and the difference in the competitive adsorption between the CO and O_2_ gas molecules on the coal surface was analyzed from a microscopic perspective using the grand canonical ensemble Monte Carlo simulation. The results showed a delayed CO release phenomenon in the initial stage of the reaction in all the experiments, and the delayed time of CO release was negatively correlated with the temperature; the relationship between the adsorption amounts of CO and O_2_ in the molecular structure model of coal was CO > O_2_. With increasing temperature, the adsorption capacity of the two gases decreased. Under the same conditions, there was competitive adsorption of the mixture of CO and O_2_ by coal, with the adsorption capacity of CO being much greater than that of O_2_. The adsorption of CO gas molecules by coal played an inhibitory role in the release of CO gas in the initial oxidation stage. The study results are expected to help understand the CO generation characteristics in the goaf of coal seam working faces and thus prevent coal mine disasters.

## Introduction

More than 95% of coal mines in China are mined by underground mining, often exposing them to multiple hazards such as gas, fire, water damage, dust, and roofing accidents. Most underground fires are closely related to the oxidation of residual coal in the goaf^1–4^. Coal undergoes oxidation reactions to produce oxidation products such as CO, CO_2_, and water. As a product of the oxidation process, CO has become the main indicator gas for underground fire prediction and forecasting^5–7^. To ensure the safety of underground workers and avoid huge losses of equipment and property, it is necessary to understand the CO generation law in the goaf of coal seam working faces.

Scholars at home and abroad have conducted significant research on the gas generation law during the low-temperature oxidation of coal^8–10^. Jiang^11^ simulated a low-temperature spontaneous combustion experiment on coal to obtain the relationship between the CO gas production and production rate and coal sample temperature; CO production was found to increase with the increase in the coal temperature. Deng et al.^12,13^ studied the oxygen consumption rate of coal samples with different particle sizes in the low-temperature oxidation stage and the corresponding relationship between the gas products and temperature using a temperature-programmed experimental device for coal spontaneous combustion; the critical temperatures of coal samples with different particle sizes were obtained. For samples taken from the Changping coal mine, Li et al.^14–16^ conducted closed oxygen consumption experiments and plotted the coal oxygen concentration and CO concentration curves with time at temperatures ranging from 293.15 to 333.15 K during the latent period of spontaneous combustion. The oxygen consumption and CO generation rates of coal at different temperatures were obtained by fitting and analyzing the oxygen concentration and CO concentration change curves, and the oxygen concentration attenuation coefficients and CO concentration growth coefficients were also obtained. Most existing studies considered the coal oxidation process as a whole, and there are few dedicated studies on the release characteristics of CO in the initial stage of the low-temperature oxidation of coal.

In recent years, many scholars have used molecular simulations to study gas adsorption by coal^17–22^. The molecular simulation method can be characterized as a method in between a macroexperiment and a microquantum chemistry method; it can help express the macrocharacteristics with a large number of microaverage values and can also be used to study a macromolecular system containing thousands of atoms^23–25^. Gensterblum^19^ developed a molecular concept, supported by literature and empirical results, for the adsorption of CH_4_, CO_2_, and H_2_O by coal over a wide range of temperatures to better understand the interactions between the different gases and H_2_O with coal. Brochard^26^ performed molecular simulations on CH_4_ and CO_2_ adsorption with an atomistic model for coal at various temperatures and pressures representative of those found in geological reservoirs. Majewska^27^ observed unusually high affinity of bituminous coal for CH_4_ compared to CO_2_ at a pressure of 2.6 MPa and a comparable affinity at 4 MPa. A poromechanical model was presented by Nikoosokhan^28^ to describe coal seams when cleats are saturated with a miscible mixture of CH_4_ and CO_2_. Scholars have mainly focused on the adsorption of CH_4_, N_2_, and CO_2_ using competitive molecular simulation methods, while few have studied the adsorption characteristics of CO in coal.

Hence, the authors of this study selected coal samples taken from the Hongqingliang coal mine as an example to conduct multiple groups of closed oxygen consumption experiments in the temperature range of 308.15–343.15 K. The delayed characteristics of CO release were explored, the adsorption of CO and O_2_ gas molecules on the coal surface was analyzed, and the competitive differences in the different gas molecules adsorbed on the coal surface were compared. The gas adsorption capacity on the coal surface was ranked to reveal the essence of the initial reaction of the spontaneous combustion of coal.

## Methods

### Experimental methods

#### Preparation and treatment of coal samples

Coal samples taken from the Hongqingliang coal mine (nonstick coal) in Ordos were selected as the research object. The coal is the middle and lower Jurassic Yan'an Formation coal seam. The 3–1 coal seam is relatively simple in structure, black in color, glossy asphalt, jagged and angular fractures, and endogenous fractures are relatively developed. It is filled with pyrite and calcite thin film, with strip-like structure and layered structure. It is semi-dark briquette. The coal type of 3–1 coal is non-stick coal. The industrial analysis of coal quality is shown in Table 1.

**Table 1:** Industrial analysis of coal quality.

	MT%	Ad%	Vdaf%	Qnetcal/g	St,d%
Coal quality	14.50	17.41	37.13	24.67	0.36

The large experimental coal samples stripped of the surface oxide layer were crushed and screened into particles with a size range of 0.425–2.00 mm and divided into ten groups weighing 2 kg each. Finally, the samples were sealed under vacuum for further experiments.

#### Experimental system and operation

**Figure 1:**
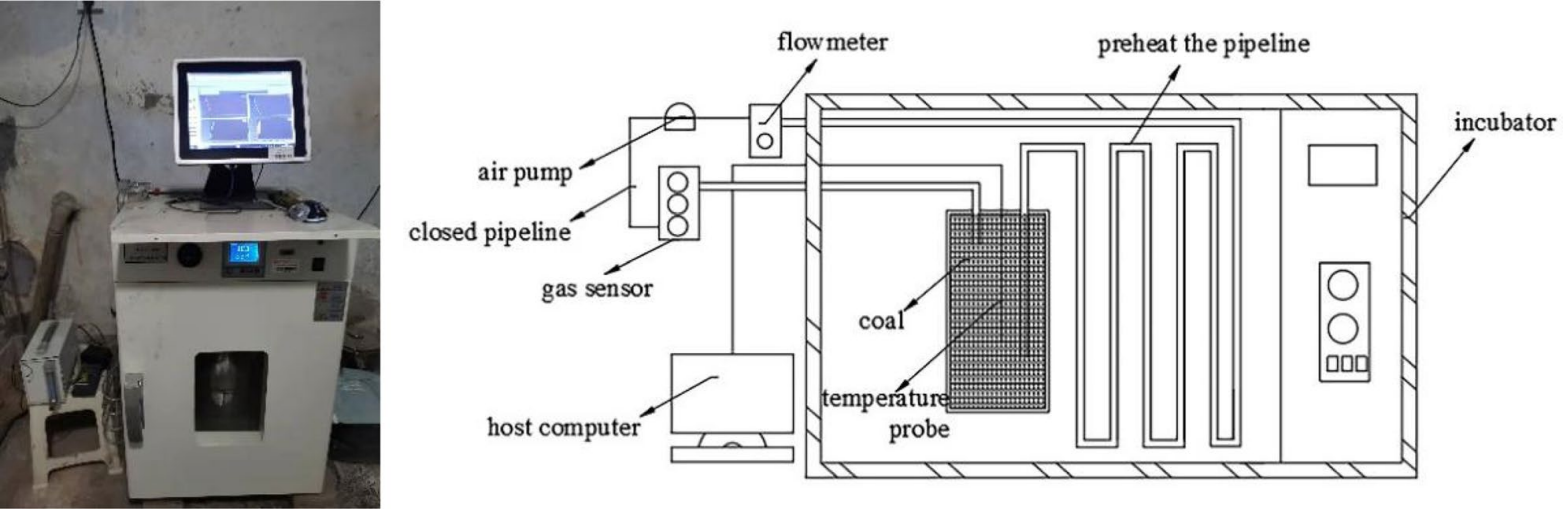
Closed oxygen consumption experimental device.

The closed oxygen consumption experiment is a method wherein the coal sample is enclosed in a container, while circulating the air inside the container through an air pump and monitoring and recording the oxygen and CO concentrations in the container in real time. Figure 1 shows the experimental device; the experimental methods have been reported in literature^14–16^. The temperature of the experimental environment was adjusted using the thermostat, and multiple groups of closed oxygen consumption experiments were conducted in the temperature range of 308.15–343.15 K. The instrument for measuring CO in this article is electrochemical CO sensor (model ZE03/CO, range 0–1000 ppm).

### Calculation details

#### Model construction

**Figure 2:**
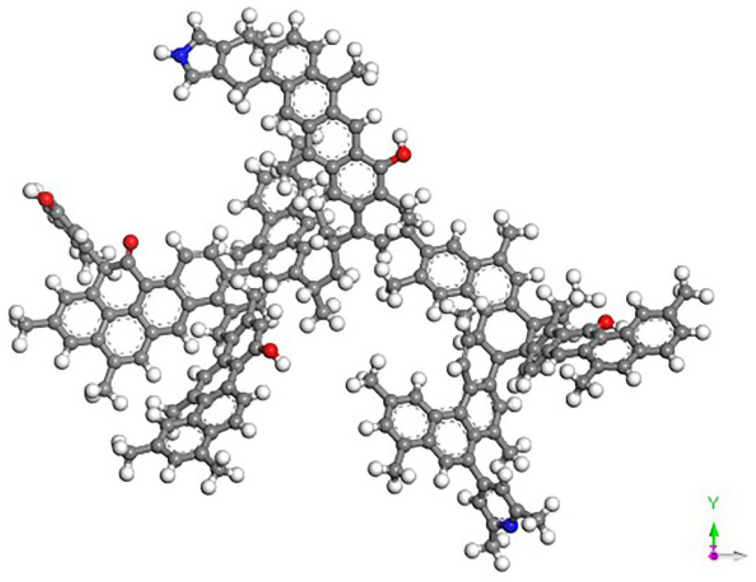
Stable configuration of the molecular structure of coal after geometric optimization.

Theoretical coal models are generally composed of coal molecules containing many atoms, and these idealized coal models are applied to solve practical problems^29–31^. The structure of coal is mainly aromatic and linked by bridging bonds, and the constituent elements are C, H, O, and N. The chemical structure model of bituminous coal molecule (C_174_H_148_O_5_N_2_) reported in literature^32^ was selected for this study; it has an aromatic backbone dominated by anthracycline and contains phenolic hydroxyl groups, carboxyl groups, pyridine, and pyrrole. Figure 2 shows the stable configuration of the molecular structure of coal after geometric optimization. Table 2 presents the model parameters.

**Table 2:** Detailed parameters of the molecular model.

Molecular formula	Molecular weight	Element content/%				Aromaticity/%
(C_174_H_148_O_5_N_2_)	2344	C	H	O	N	66.5
		89.04	6.36	3.41	1.19	

#### Calculation method

**Figure 3:**
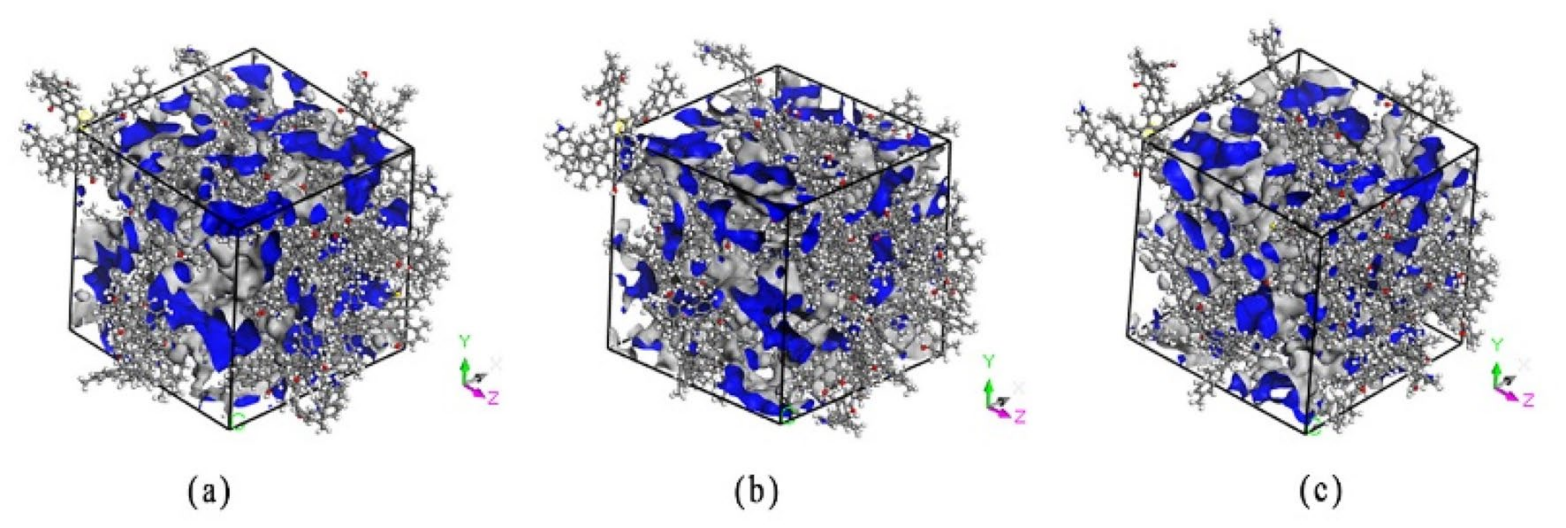
Supercell structures of coal at different temperatures: (**a**) 308.15 K; (**b**) 318.15 K; (**c**) 328.15 K.

A molecular dynamics simulation calculation was conducted using the Module Forcite and Module Sorption in Materials Studios 2018 software. The COMPASS force field widely used in coal adsorption studies was selected as the calculation force field; it uses intermolecular electrostatic and van der Waals force interactions to simulate and calculate the bonding between atoms on the coal surface^33,34^. The geometric configurations of the coal and gas molecules were optimized by Module Forcite, and the crystal cells were relaxed using molecular dynamics methods under normal temperature and pressure (NPT) system synthesis, along with temperature control. The summation of the intermolecular electrostatic and van der Waals interactions were calculated using the Ewald method and atom-based method with a cut-off radius of 1.25 nm, respectively^35–37^. The structural model of the macromolecules in the coal was obtained. After the system was optimized for equilibrium, the Module Sorption was launched under the task of adsorption isotherms to simulate the adsorption capacity of the CO and O_2_ molecules in the macromolecule model of coal at a fixed temperature to obtain the conformation of adsorption. Each simulation calculation comprised 1 × 10^7^ Monte Carlo steps, of which the first 5 × 10^6^ steps were used to balance the system and monitor the convergence curve of the system energy with time to ensure that the system reached equilibrium. The last 5 × 10^6^ steps were calculated for the output of the results of the thermodynamic parameters such as the adsorption capacity, heat of adsorption, and adsorption energy. For the simulation calculation, the pressure (0.01–10 MPa) and temperature (308.15, 318.15, and 328.15 K) were set. Figure 3 shows the super cell structure of coal at three temperatures: (a) 308.15 K; (b) 318.15 K; (c) 328.15 K. At the three temperatures, the free volumes of a molecular unit cell were 12,872.69, 12,732.29, and 12,030.33 Å^3^, respectively. The temperature had a significant effect on the molecular unit cell structure of coal.

## Results and discussion

### Analysis of delay in CO release

**Figure 4:**
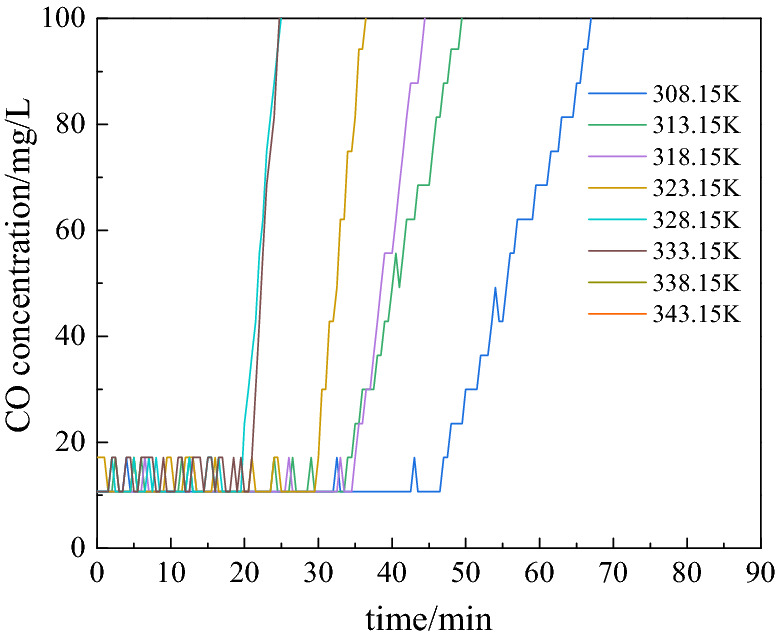
CO release concentration in the initial stage of the experiment.

The data of the CO release concentration in the initial stage of the oxidation reaction for each group of experimental coal samples were analyzed. As shown in Fig. 4, the CO release concentrations of each group of samples start to increase rapidly in the range of 10.7066–17.1306 mg/L in the initial reaction stage after the continuous fluctuation in the approximate horizontal state. In this study, the time of continuous fluctuation in the CO concentration near the level in the initial reaction stage was called the “delay time of CO release.” This delay time varies for different experimental temperatures.

**Figure 5:**
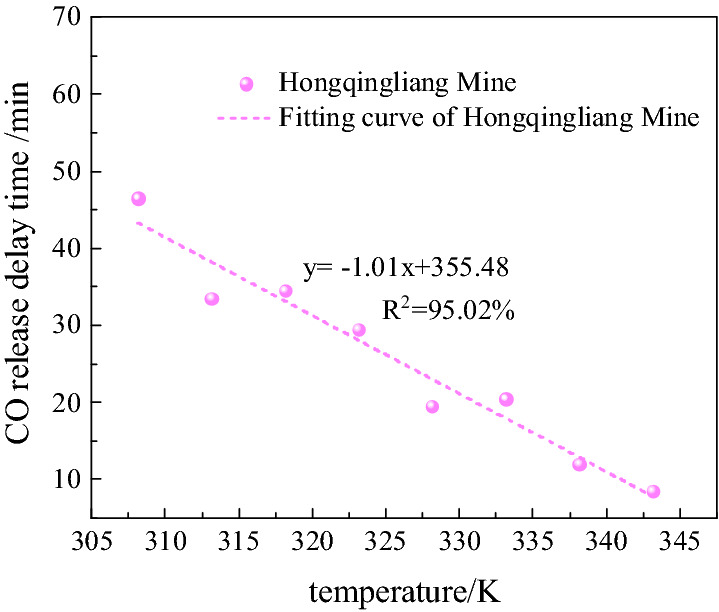
Fitting curve for mine sample.

The data before 100 min for each group of coal samples taken from the Hongqingliang mine were analyzed, and the delay time of the CO release was counted for each group of experiments. Table 3 presents the delay time of the CO release for each group of experiments. The analysis of the data, presented in Table 3, shows a delay in the CO release in the initial stage of the low-temperature oxidation of coal, and as the temperature increases, the delay time decreases. At 308.15 K, the delay time of CO release was 46.5 min; at 343.15 K, the time was 8.5 min; in the temperature range of 308.15–343.15 K, the delay time decreased by 81.72%. Figure 5 shows the fitted curves of the two factors above mentioned. As shown in Fig. 5, the delay time of the CO release is linearly related to the temperature, which means that the delay time of the CO release decreased linearly with the increase in the temperature.

**Table 3:** Delay time statistics of CO release.

Coal samples	Variable	Experimental temperature/K							
		308.15	313.15	318.15	323.15	328.15	333.15	338.15	343.15
Hongqingliang coal mine	Delay time of CO release/min	46.5	33.5	34.5	29.5	19.5	20.5	12	8.5
	Concentration of initial point/mg/L	10.70	10.70	10.70	10.70	10.70	10.70	10.70	10.70

### Difference in competitive adsorption of gases by coal

#### Adsorption isotherm

**Figure 6:**
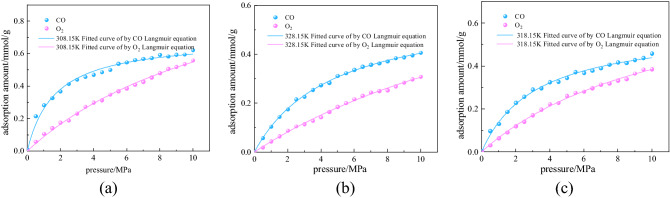
Adsorption isotherms of CO and O_2_ at different temperatures.

Figures 6(a)–(c) show the variation curves of the adsorption capacity of CO and O_2_ for each single-component gas on the surface of the macromolecules in coal with respect to the pressure at temperatures of 308.15, 318.15, and 328.15 K. The adsorption capacity of coal for both CO and O_2_ increased first and then stabilized, conforming to the Langmuir model. Under the same conditions, the adsorption of CO was greater than that of O_2_, indicating that the interaction force with CO was greater than that between coal and O_2_. Under the same temperature and pressure, the delay in the CO release appeared in the closed oxygen consumption experiment because of the stronger adsorption capacity of CO for coal. In addition, comparing the CO adsorption capacities at different temperatures, it can be found that the amount of adsorption decreased, and the adsorption capacity weakened with increasing temperature. This shows that the delay time of CO release was negatively correlated to the temperature in the experiments. Table 4 shows the Langmuir fitting parameters of the single-component adsorption at different temperatures.

**Table 4:** Langmuir fitting parameters of single-component adsorption at different temperatures.

Temperature/K	Gas	a	b	R^2^
308.15	CO	0.69752	0.59819	0.98653
	O_2_	1.30745	0.07236	0.99685
318.15	CO	0.57922	0.31159	0.99901
	O_2_	0.83309	0.08563	0.99715
328.15	CO	0.59511	0.21244	0.99899
	O_2_	0.97741	0.04579	0.99709

**Figure 7:**
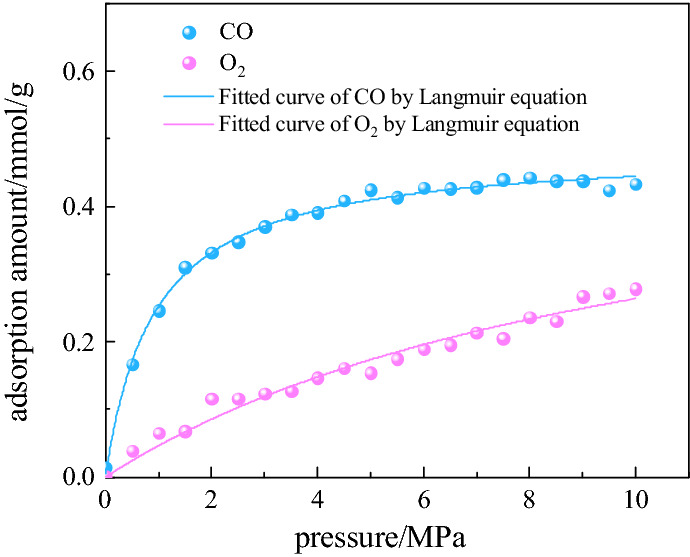
Competitive adsorption equilibrium isotherms of CO/O_2_ mixture on coal at 308.15 K.

Figure 7 shows the variation curves of the adsorption capacity of CO and O_2_ mixture (with a molar ratio of 1:1) on the coal surface with respect to the pressure at a temperature of 308.15 K. As shown, there is competitive adsorption of the CO and O_2_ mixture for coal under the same conditions, and the adsorption of CO is approximately twice as much as that of O_2_, consistent with the law shown in Fig. 6. Table 5 shows the Langmuir fitting parameters of the competitive CO/O_2_ adsorption at a temperature of 308.15 K. This further proves that the delayed release of CO is due to the higher adsorption capacity of coal for CO than for O_2_.

**Table 5:** Langmuir fitting parameters of competitive CO/O_2_ adsorption at 308.15 K.

Temperature/K	Gas	a	b	R^2^
308.15	CO	0.48587	1.07823	0.99467
	O_2_	0.55148	0.09192	0.96845

#### Heat of adsorption

The heat of adsorption is the heat released by the adsorbed gas during its adsorption by coal. It can accurately describe the physicochemical properties and the adsorption capacity of the coal in the adsorption process. Although it is difficult to measure accurately using experimental methods, it can be easily measured using molecular simulation methods. Table 6 presents the equivalent heat of adsorption of the CO and O_2_ gases under different pressure and temperature conditions. For the same gas, the effects of temperature and pressure variations on the equivalent heat of adsorption are low and largely constant in the range of the simulated temperature and pressure. At 308.15 K, the average heats of adsorption of the single-component CO and O_2_ by coal are 24.32 and 12.44 kJ mol^−1^, respectively, which shows that the adsorption capacity of coal for the two gases follows the rule CO > O_2_, and the same is true at the rest of the temperatures. At the same temperature, the equivalent heat of adsorption of CO was approximately twice that of O_2_. The highest equivalent heat of adsorption was 25.3633 kJ mol^−1^, which is less than 42 kJ mol^−1^, and it can also be determined that both CO and O_2_ underwent physical adsorption by coal at this time.

**Table 6:** Equivalent heat of adsorption of single-component CO and O_2_ at different pressures and temperatures.

Pressure/MPa	Equivalent heat of adsorption of CO/kJ/mol			Equivalent heat of adsorption of O_2_/kJ/mol		
	308.15 K	318.15 K	328.15 K	308.15 K	318.15 K	328.15 K
0.01	23.91	22.55	21.72	12.73	11.36	11.03
0.5	24.82	22.78	22.32	12.13	11.47	11.52
1	24.58	22.92	22.72	12.36	11.54	11.84
1.5	23.72	24.05	22.14	12.31	11.81	11.62
2	22.71	23.44	22.53	12.40	11.71	11.88
2.5	23.42	23.36	22.43	12.35	11.67	11.74
3	24.25	23.84	23.20	12.21	11.64	12.31
3.5	24.26	24.17	22.78	12.58	12.14	11.78
4	23.97	23.84	23.49	12.53	11.69	12.08
4.5	24.18	23.81	23.26	12.52	11.81	11.69
5	24.33	23.81	22.35	12.30	12.08	12.03
5.5	24.29	23.54	23.31	12.38	11.69	12.06
6	24.37	23.84	23.75	12.27	11.53	12.20
6.5	24.63	23.72	23.08	12.54	12.00	11.82
7	24.13	23.46	23.10	12.62	11.69	12.03
7.5	24.79	23.68	23.59	12.44	12.01	11.74
8	24.36	23.76	23.69	12.26	11.96	12.09
8.5	24.94	24.02	23.41	12.42	12.04	11.82
9	24.41	24.00	23.55	12.66	11.79	11.99
9.5	24.93	23.97	23.66	12.44	12.24	11.83
10	24.82	23.84	23.28	12.72	12.07	11.79

Many scholars have done a lot of research on the adsorption of coal with CO and O_2_ gas molecules. Wenhu^38^ studied the adsorption performance of coal for CO gas. Through the analysis and calculation of experimental data, it was concluded that the adsorption heat of CO by different coal samples is between 12–25 kJ/mol, and the adsorption form of coal for CO is mainly physical adsorption. The adsorption heat of CO obtained in this paper is 21.74–24.94 kJ/mol, which is in good agreement with the experimental results. Some scholars have studied the adsorption performance of coal for O_2_ by GCMC simulation, and obtained the adsorption heat of oxygen in the range of 8–17.30 kJ/mol^39–41^. The adsorption heat of O_2_ obtained by this simulation is 11.03–12.73 kJ/mol. The simulation results in this paper are within its scope, which verifies the correctness of the simulation in this paper.

#### Interaction energy and energy distribution

**Figure 8:**
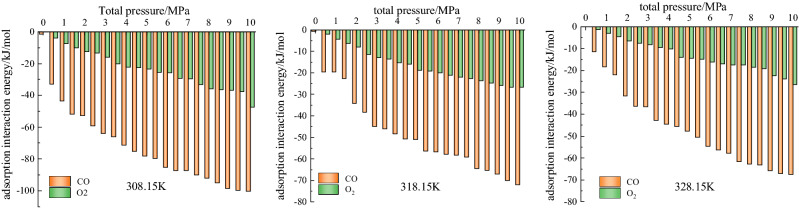
Functional relationship between the single-component gas adsorption energy and total pressure at different temperatures.

In this study, the total energy is defined as the sum of the interaction energies in the configurations of the adsorbate and adsorbent and internal energy of the adsorbate molecules. The greater the interaction energy, the more likely the adsorption to occur, indicating a more stable system^40^. As shown in Fig. 8, the interaction energy between coal and CO is greater than that between coal and O_2_ at the same temperature, indicating that coal preferentially adsorbs CO, making the adsorption of CO greater than that of O_2_, consistent with the results shown in Fig. 7.

**Figure 9:**
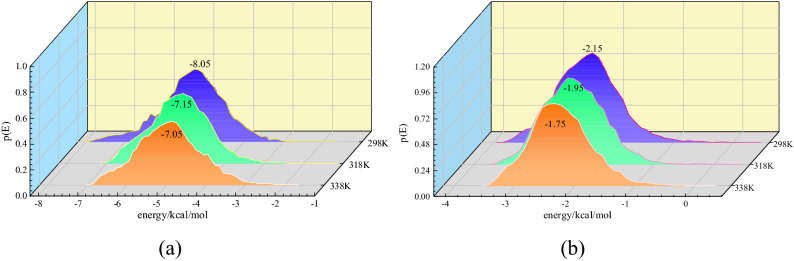
Energy distributions of two gases adsorbed by coal (**a**) CO; (**b**) O_2_.

In addition to the macroscopic phenomena of the adsorption volume and heat of adsorption, the molecular-scale adsorption sites and the probability distribution of the potential energy can be obtained using Monte Carlo simulation. Figure 9 shows the energy distributions of CO and O_2_ for the adsorption process at temperatures of 308.15, 318.15, and 328.15 K. For the same gas, only one peak can be seen in the energy distribution curve at each temperature, indicating the existence of an interaction force between the two gases and the coal, namely the van der Waals force. The peak of the energy curve represented the maximum gas adsorption at that energy. As shown in Figs. 9(a) and (b), the energy corresponding to the initial stage of adsorption is different. The lower the initial energy, the greater the adsorption potential in the coal pores^42^. In Fig. 9, the initial energies corresponding to CO and O_2_ at 308.15 K are − 8.05 and − 3.95 kJ mol^−1^, respectively, and the initial energy corresponding to CO is lower. This indicates that the CO gas molecules were more easily adsorbed by the micropores of the coal under the adsorption potential, as was the case at the rest of the temperatures.

## Conclusions

To study the release law of CO in coal under low-temperature oxidation, the delayed release law of CO by coal was investigated by conducting closed oxygen consumption experiments and the grand canonical ensemble Monte Carlo simulation (GCMC). The following conclusions can be drawn:

1. The delayed release of CO was observed in the initial stage of the low-temperature oxidation of coal. The delayed time of CO release was exponentially distributed with the temperature. The delayed time of CO release decreased with the increase in the temperature in the range of 308.15–353.15 K.
2. The adsorption isotherms of CO and O_2_ were in good agreement with the Langmuir adsorption isotherm model, observed by analyzing the competitive adsorption laws of CO and O_2_ using the Materials Studios software. The relationship between the adsorption capacity of the two gases in the coal structure model was CO > O_2_. In the temperature range of 308.15–328.15 K, the temperature rises to inhibit gas adsorption. At the same temperature and pressure, there is competitive adsorption of the mixed gas of CO and O_2_ by coal, and the adsorption capacity of CO is evidently higher than that of O_2_. The equivalent heat of adsorption and interaction energy of CO were much greater than that of O_2_, indicating that the adsorption capacity of the molecular structure model of coal for CO was significantly greater than that for O_2_.
3. In the initial stage of low-temperature oxidation, coal and oxygen reacted to produce CO gas; however, it was not detected. This was because the adsorption of the CO gas molecules inhibited the release of CO. Therefore, when CO gas is selected as an indicator gas to study the spontaneous combustion of coal, its adsorption capacity should be analyzed so that the coal–oxygen reaction can be obtained more accurately.
